# Prenatal exposure to hypoxic risk conditions in autistic and neurotypical youth: Associated ventricular differences, sleep disturbance, and sensory processing

**DOI:** 10.1002/aur.3250

**Published:** 2024-10-16

**Authors:** Cristian Preciado, Maria Baida, Yi Li, Yan Li, Carly Demopoulos

**Affiliations:** ^1^ Department of Psychiatry and Behavioral Sciences University of California San Francisco San Francisco California USA; ^2^ Department of Radiology & Biomedical Imaging University of California San Francisco San Francisco California USA; ^3^ Present address: University of Arizona Tucson Arizona USA

**Keywords:** autism spectrum disorder, freesurfer, neurodevelopment, prenatal hypoxia, sensory processing, sleep disturbance, thalamus, third ventricle

## Abstract

There is a growing body of research that suggests conditions during the period of pregnancy and birth can affect how autism spectrum disorder (ASD) presents itself. This study aimed to investigate the incidence of oxygen deprivation during this period known as prenatal and perinatal hypoxic risk (HR) conditions in ASD compared with neurotypical control (NTC) youth. We also examined ventricular morphology variations associated with HR exposure, and to evaluate associations with clinical symptoms. Results from a cohort of 104 youth revealed a higher incidence of exposure to prenatal hypoxic conditions in the ASD group. Additionally, ASD individuals with prenatal hypoxic exposure (ASD + HR) demonstrated larger third ventricle volumes compared with both ASD and NTC individuals without such exposure (ASD‐HR and NTC‐HR, respectively). Furthermore, associations were identified between prenatal hypoxic exposure, third ventricle volume, sensory dysfunction, and severity of sleep disturbances. These findings suggest exposure to prenatal hypoxic risk conditions may exacerbate or modify the neurodevelopmental trajectory and symptom severity in ASD, emphasizing the need for better prenatal care and specific interventions to reduce these risks.

## Lay Summary

In our study, we looked into how a lack of oxygen during pregnancy and birth, known as hypoxic risk conditions, can affect the development of children. We found that youth with autism spectrum disorder (ASD) were more likely to have been exposed to these conditions compared with youth without autism. This lack of oxygen may be associated with third ventricle enlargement and clinical symptoms, such as problems with sleep and sensory processing. Our findings emphasize the need for better prenatal care that reduces these risks.

## INTRODUCTION

Autism spectrum disorder (ASD) is a complex developmental condition that manifests in early childhood. ASD is defined by deficits in social communication and restricted and repetitive patterns of behaviors and interests, and it is characterized by early developmental onset (American Psychiatric Association, 2013). Both genetic and environmental factors have been associated with ASD. The environmental factors may present in the prenatal—referring to the period before birth—and/or perinatal period, which relates to the period around the time of birth (Gardener et al., 2009; Hisle‐Gorman et al., 2018; Kinney et al., 2008; Lu et al., 2022), and may include medical conditions, psychosocial support, teratogen or other environmental exposures, advanced maternal and paternal age, medication use, and lifestyle factors, such as diet and stress (Lord et al., 2018; Ornoy et al., 2015; Seebeck et al., 2023). These environmental factors are of particular interest, since they can potentially affect postnatal neurodevelopment. Additionally, they have been associated with poor sleep, learning difficulties, and autism severity (Chien et al., 2019; Ornoy et al., 2015; Pesonen et al., 2009).

Prenatal hypoxia—an insufficient supply of oxygen during periods of prenatal development—is a risk factor for brain injury and is associated with effects on brain structure and function, including problems with learning and memory and impact on gene expression leading to neurodegeneration later in life (Nalivaeva et al., 2018). In a twin study of 194 monozygotic and dizygotic pairs, Froehlich‐Santino et al. (2014) found that respiratory distress and other markers of hypoxia were strongly associated with an increased likelihood of ASD. Given these findings, prenatal hypoxic exposure may be associated with a specific phenotype of clinical symptom presentation in ASD.

Kingdom and Kaufmann (1997) describe prenatal and perinatal risk factors that may potentially induce hypoxia throughout pregnancy (e.g., preeclampsia, intrauterine growth restriction (IUGR), placental insufficiency, nuchal cord complications, etc.). By following placental blood flow disturbances, the authors broke down hypoxic risk conditions into three stages: preplacental; uteroplacental; postplacental. In preplacental hypoxia there is insufficient oxygenation of maternal blood thereby inducing hypoxic risk conditions in the mother, placenta, and fetus. In uteroplacental hypoxia there is restricted blood entry into uteroplacental tissues thereby inducing hypoxic risk conditions in the placenta and fetus. Lastly, in postplacental hypoxia there is a major defect in fetal‐placental perfusion thereby inducing hypoxic conditions in only the fetus (Hutter et al., 2010). This categorization is important for understanding specific hypoxic risk conditions and allows for a more nuanced description of their subsequent impact on fetal development. For instance, placental patterns of injury, such as maternal vascular malperfusion (MVM) or venous rupture can be related to perfusion altering conditions, such as preeclampsia, a uteroplacental hypoxic risk condition (Redline, 2022). Nuchal cord—the wrapping of the umbilical cord around the fetal neck—has been found to have occurred with a higher frequency among children with autism, is considered a postplacental hypoxic risk condition (Peesay, 2017).

The most metabolically active areas of fetal and neonatal brains are most sensitive and vulnerable to hypoxic injury, and are also most fundamental to neurologic function. Thus, while hypoxia alone does not define the pathogenesis of ASD, its presence during critical periods of brain formation may alter the trajectory of development, with impact on clinical symptom presentation. Indeed, a study by Tunç et al. (2019) compared developmental changes in anatomical (cortical thickness, surface area, and volume) and diffusion metrics (fractional anisotropy and apparent diffusion coefficient) between children with ASD (*n* = 247) and typically developing children (TDC) (*n* = 220) and found that the difference between chronological age and brain age correlated with ASD symptom severity. Specifically, those with increased symptom severity had a delayed brain age. Additionally, prenatal hypoxia has been demonstrated to lead to structural alterations, such as reduced brain volume, thinning cortex, and reduced connectivity, which are associated with neurodevelopmental deficits (Miller et al., 2016).

In particular, the ventricles and subventricular zones (SVZs) are vulnerable to hypoxic risk conditions, particularly in premature infants (Pașca et al., 2019). De Vries and Groenendaal (2010) have further described this susceptibility by detailing distinct patterns of brain injury in full‐term neonates following hypoxic events, such as ventricular abnormalities linked to periventricular white matter regions.

Despite these findings, there is a gap in understanding how prenatal hypoxic risk conditions specifically affect ventricular brain structures in individuals with ASD. Further, enlargement of the third ventricle and resulting atrophy of surrounding brain regions, such as the thalamus and hypothalamus, are responsible for regulating essential body functions, including sleep and sensory processing (Wolfe et al., 2015). Therefore, we aimed to investigate the following:

1. The incidence of exposure to prenatal hypoxic risk conditions in youth with ASD compared with neurotypical controls (NTCs).
2. Differences in ventricular morphology among autistic youth with exposure to prenatal hypoxic risk conditions (ASD + HR), those without exposure (ASD‐HR), and NTCs with (NTC + HR), and without hypoxic risk conditions (NTC‐HR).
3. Associations between the number of hypoxic risk conditions, ventricular volume, sleep problems, autism symptom severity, and sensory processing.

To evaluate these aims, we utilized the classifications of hypoxic risk conditions and placental perfusion stages defined in previous work (Hutter et al., 2010; Kingdom & Kaufmann, 1997). Understanding the sequelae associated with prenatal hypoxic risk is crucial for early identification and monitoring of at‐risk individuals and developing targeted interventions to mitigate the adverse effects of prenatal hypoxia.

## METHODS

### Participants

A case–control study design was employed involving 104 youth with ASD (*n =* 69; *n* = 42 male, *n* = 27 female) and NTC (*n =* 35; *n* = 17 male, *n* = 18 female). Participants had a mean age of 12.46 years. Participants were recruited from the University of California San Francisco (UCSF) clinics and local advertisements. All procedures were approved by the UCSF Institutional Review Board. Data were anonymized for analysis. Participants provided their assent, and consent was obtained from parents or legal guardians prior to enrollment. Participant demographics are presented in Table 1. Diagnosis of ASD was confirmed by a licensed clinical psychologist who had achieved research reliability on the Autism Diagnostic Observation Schedule‐2 (ADOS‐2; Lord et al. (1989)) and the Autism Diagnostic Interview‐Revised (ADI‐R; Lord et al. (1994)).

**TABLE 1:** Participant demographics.

	ASD (*n* = 69)		NTC (*n* = 35)	
Age (*M*, SD)	12.60	2.72	12.46	3.03
Range	[8.08–17.33]		[8.42–16.83]	
Sex at birth (*n*, %)				
Male	42	61%	17	49%
Female	27	39%	18	51%
Race				
Black	1	1%	0	0%
Asian	10	15%	6	17%
Multiracial	19	28%	13	37%
White	38	55%	16	46%
Native American	1	1%	0	0%
Ethnicity				
Hispanic	18	26%	4	11%
Hypoxic risk (HR)				
(+)HR	36	52%	5	14%
(−)HR	33	48%	30	86%

*Note*: (+)HR, positive for at least one hypoxic risk condition; (−)HR, no reported history of prenatal hypoxic risk conditions.

### Measures

#### Hypoxic risk conditions

Parents completed a detailed developmental questionnaire that included questions about birth history and prenatal and postnatal conditions—(e.g., cyanosis, preeclampsia, etc.). Parents were able to type into a free text field to list any and all prenatal and postnatal conditions and complications. Hypoxic risk conditions were classified into preplacental, uteroplacental, and postplacental hypoxic risk conditions, as defined by Hutter et al. (2010), and Kingdom and Kaufmann (1997). Preplacental hypoxic risk conditions included chronic maternal pulmonary disease, maternal heart disease, hematological disorders, and infections that might lead to insufficient oxygenation of maternal blood, impacting both the mother and fetus. Uteroplacental hypoxia was defined by complications that restrict maternal blood entry into uteroplacental tissues, such as preeclampsia and its associated HELLP syndrome symptoms. IUGR with preserved end‐diastolic flow velocity, abnormal placentation, uterine hemorrhage, and other signs of placental insufficiency also were considered uteroplacental hypoxic risk conditions. Finally, postplacental hypoxic conditions were conditions in which maternal blood entered the intervillous space at a normal or reduced rate, but defects in fetoplacental perfusion caused the fetus to experience hypoxia. Fetal heart diseases, fetal placental vascular obstruction, including nuchal cord complications, and fetal hypoxia‐mediated complications including meconium aspiration, metabolic and hematologic disturbances, and cyanosis were considered postplacental hypoxic risk conditions. The conditions documented by parents on the developmental history questionnaire were summarized and classified according to these criteria.

#### Sleep disturbance

We used the Children's Sleep Habits Questionnaire (CSHQ) to assess for sleep problems among the participants (Owens et al., 2000). There are a total of 33 questions and each addresses a specific sleep behavior, with parents rating the frequency of occurrence over the past week on a three‐point scale: “Usually” (5 or more times per week), “Sometimes” (2–4 times per week), and “Rarely” (never or 1 time per week). This is further categorized into eight subscales: bedtime resistance, sleep onset delay, sleep duration, sleep anxiety, night wakings, parasomnias, sleep disordered breathing, and daytime sleepiness. The questionnaire yields a total score and subscale scores, with higher scores indicating more severe sleep disturbances.

#### Autism symptom severity/sensory dysfunction

The Social Communication Questionnaire (SCQ; Rutter Bailey & Lord, 2003) was administered to parents of participants to assess autism symptom severity. The SCQ generates a total score, with higher values indicative of endorsement of more symptoms. The Child/Adolescent Sensory Profile‐2nd Edition (SP‐2; Dunn (2014)) was also administered to parents to quantify sensory processing behaviors. The four quadrant scores describe the degree of differences in sensory processing, including sensory seeking, sensory avoiding, sensory sensitivity, and low registration of sensory information, with higher scores indicating that participants experience more of these symptoms than others and lower scores indicating less of these symptoms than others.

#### Magnetic resonance image acquisition and processing

Magnetic resonance images (MRIs) were acquired with a 3T MR scanner (GE Healthcare, Waukesha, WI). T1‐weighted sequence was acquired for tissue segmentation using TR = 6.95 ms, TE = 2.92 ms, TI = 1060 ms, Thickness = 1 mm, field‐of‐view [FOV] = 256 mm, and matrix size = 256 × 256. T1 Images were visually inspected for motion artifacts (initial *N* = 135) and 15 images were removed due to significant image degradation associated with participant motion, resulting in *N* = 120. Notably The T1 images were then processed through Freesurfer segmentation software Version 7.4.0; (Fischl, 2012) to segment the lateral, third, and fourth ventricles. Qoala‐T software Version 1.2.1 (Klapwijk et al., 2019), a supervised machine‐learning tool, was used to assess the quality of segmentation using a random forest analysis. Images with Qoala‐T scores below 30 were rejected, resulting in *N* = 106. Images with scores above 70 passed, while those scoring between 30 and 70 were further visually inspected for proper segmentation. Images visually inspected for proper segmentation were assessed following the manual quality control procedure developed by Klapwijk et al. (2019). Images failing to meet quality criteria were excluded from analysis resulting in a final sample size of *N* = 104. Ventricular volume and total intracranial volume were computed from the segmented images.

#### Statistical analysis

T1 image segmentations were measured relative to estimated total intracranial volume, age, and sex at birth to account for differences in head size according to best practices as outlined by Backhausen et al. (2021). SPSS Statistics Version 29 (IBM Corp., 2023) was used to compute statistical tests. A Chi‐Square Test of independence was performed to determine if there were group differences in exposure to prenatal hypoxic risk conditions in the ASD group and NTC group. Next, a Quade nonparametric analysis of covariance (ANCOVA) was performed to investigate group differences (ASD + HR, ASD‐HR, NTC + HR, NTC‐HR) in ventricular volume. Due to the nonnormality of data, spearman correlations were computed between the CSHQ data and the number of exposures to prenatal hypoxic risk conditions to evaluate the association between the severity of sleep disturbances and hypoxic risk. Nonparametric correlations were computed for the overall sample and individual groups. R version 4.3.1 (R Core Team, 2023) was used with the packages ggplot2 version 3.5.1 (Wickham, 2016), dplyr version 1.1.4 (Wickham et al., 2019) for the creation of visualizations for the group differences in exposure to prenatal hypoxic risk conditions, group differences in ventricular volume, and scatterplots demonstrating associations between variables.

We ensured that all assumptions for statistical tests were met before analysis. Bonferroni corrections were applied to control for the risk of Type I error due to multiple comparisons.

#### Visualization of differences in ventricular volume

To visualize spatial differences in ventricular volume, voxel‐wise subtraction was applied on composite images made from the segmented images of groups with significant differences in ventricular volume similar to the process as described in Hassen et al. (2016). Composite images were made using Freesurfer's “make_average_subject” and aligned to talairach coordinates before subtraction. Composite segmentations were extracted using R package oro.nifti version 0.11.4 (Whitcher et al., 2011). Spatial differences were defined as the difference between the ASD + HR composite group image and nonhypoxic composite group images (ASD‐HR, NTC‐HR).

## RESULTS

### Group differences in exposure to prenatal hypoxic risk conditions

**FIGURE 1:**
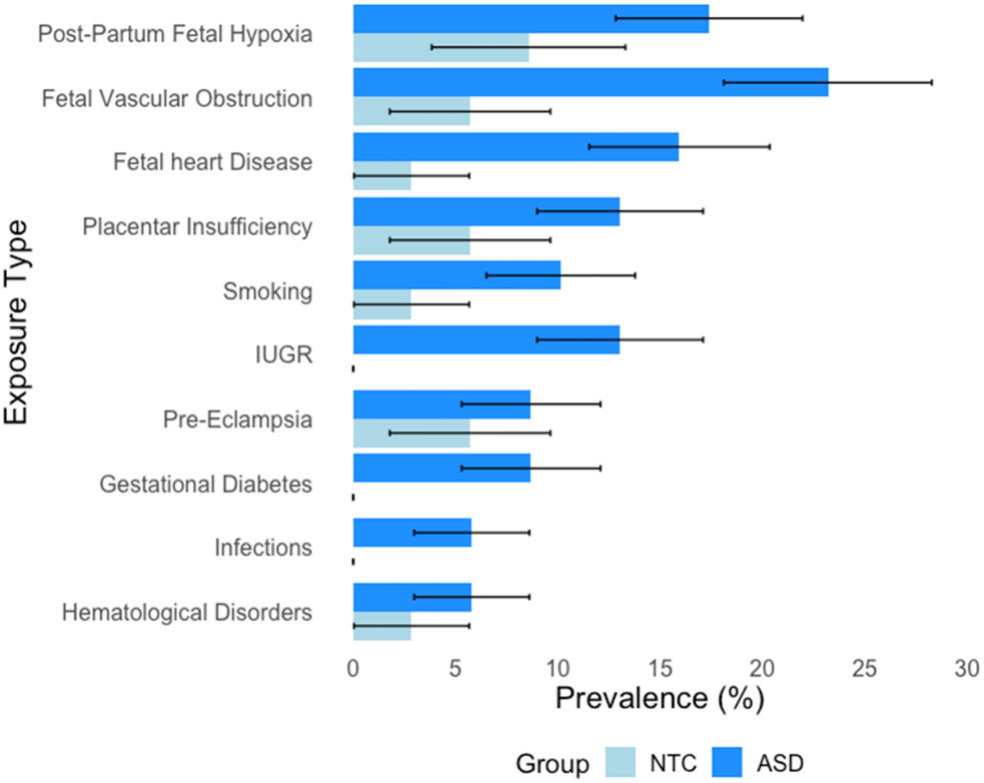
Exposure prevalence to hypoxic risk conditions by group. Proportions represent the percentage of participants exposed to different types of hypoxic risk conditions in the autism spectrum disorder (ASD) and neurotypical control (NTC) groups. Participants may be represented in more than one category if they had multiple exposures.

There were 36 participants in the ASD group with prenatal exposure to at least one hypoxic risk condition, whereas only 5 NTC participants had history of any prenatal hypoxic risk. This difference in incidence of prenatal hypoxic risk exposures was statistically significant, with $\chi^{2}$ (1, *N* = 104) = 13.96 value, *p* < 0.001. The number of exposures for each group is presented in Table 2, and the prevalence of exposures is in Figure 1.

**TABLE 2:** Hypoxic risk conditions criteria by placental stage in sample.

Type	Number of exposures by group		
	Total	ASD	NTC
Preplacental			
Gestational diabetes	6	6	0
Maternal hematological disorders	5	4	1
Infections	4	4	0
Uteroplacental			
Preeclampsia	8	6	2
Intrauterine growth restriction (IUGR)	9	9	0
Smoker	8	7	1
Placental insufficiency	11	9	2
Postplacental			
Fetal heart disease	12	11	1
Fetal placental vascular obstruction	18	16	2
Fetal hypoxia‐mediated complications	15	12	3

*Note*: The table presents criteria and number of exposures for each prenatal hypoxic risk condition broken down by placental stage. Exposures are not exclusive participants.

### Group differences in ventricular volume

**FIGURE 2:**
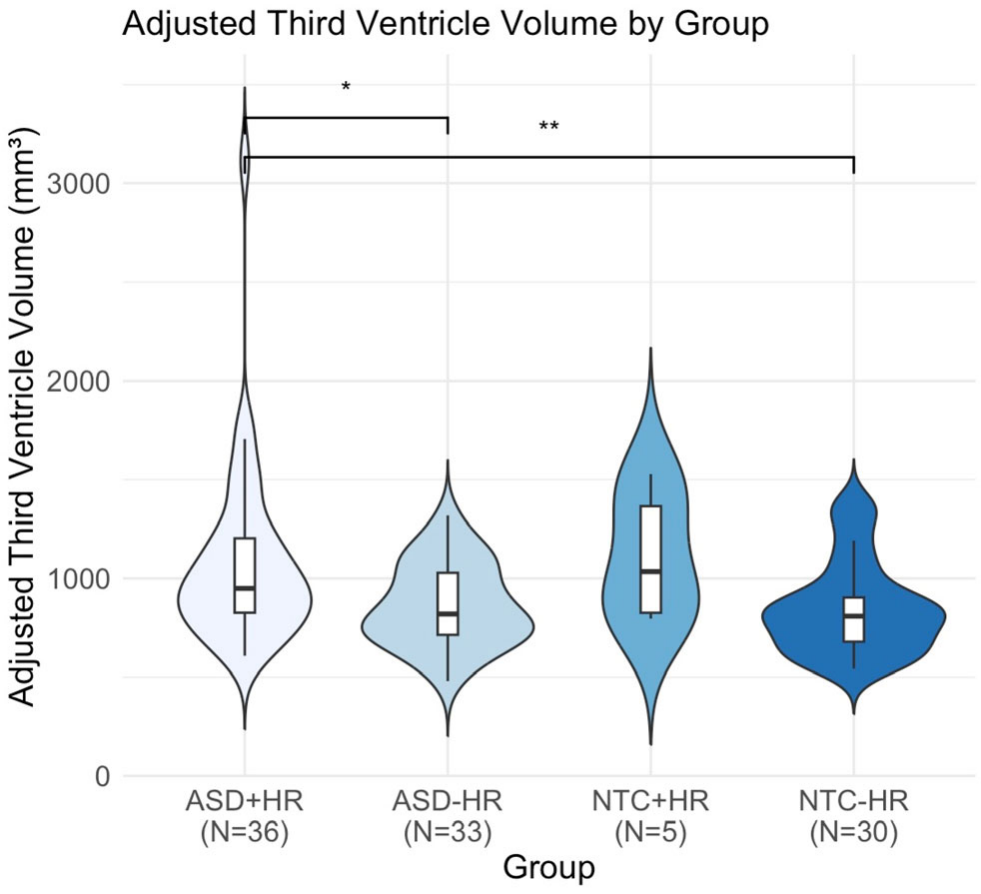
Adjusted third ventricle volume by group. Third ventricle volume was adjusted by estimated total intracranial volume, age, and sex at birth. *p = 0.032, **p = 0.009. ASD, autism spectrum disorder; HR, hypoxic risk; NTC, neurotypical control.

A significant main effect of hypoxic risk was identified for the volume of the third ventricle among subject groups with and without hypoxic risks, ASD + HR, ASD‐HR, NTC + HR, NTC‐HR; (Quade nonparametric ANCOVA test: *p* = 0.027). Pairwise comparisons demonstrated that the ASD + HR group had significantly larger third ventricle volumes compared with the ASD‐HR group (*t*(100) = 2.16, *p* = 0.032) and to the NTC‐HR group, *t*(100) = 2.680, *p* = 0.009. No significant differences were observed when comparing the NTC + HR group with the other groups; however, with *N* = 5 for this group, statistical power was not adequate to interpret those contrasts. See Figure 2 for a violin plot of third ventricle volume by group. No significant group differences were identified in other ventricular structures.

**FIGURE 3:**
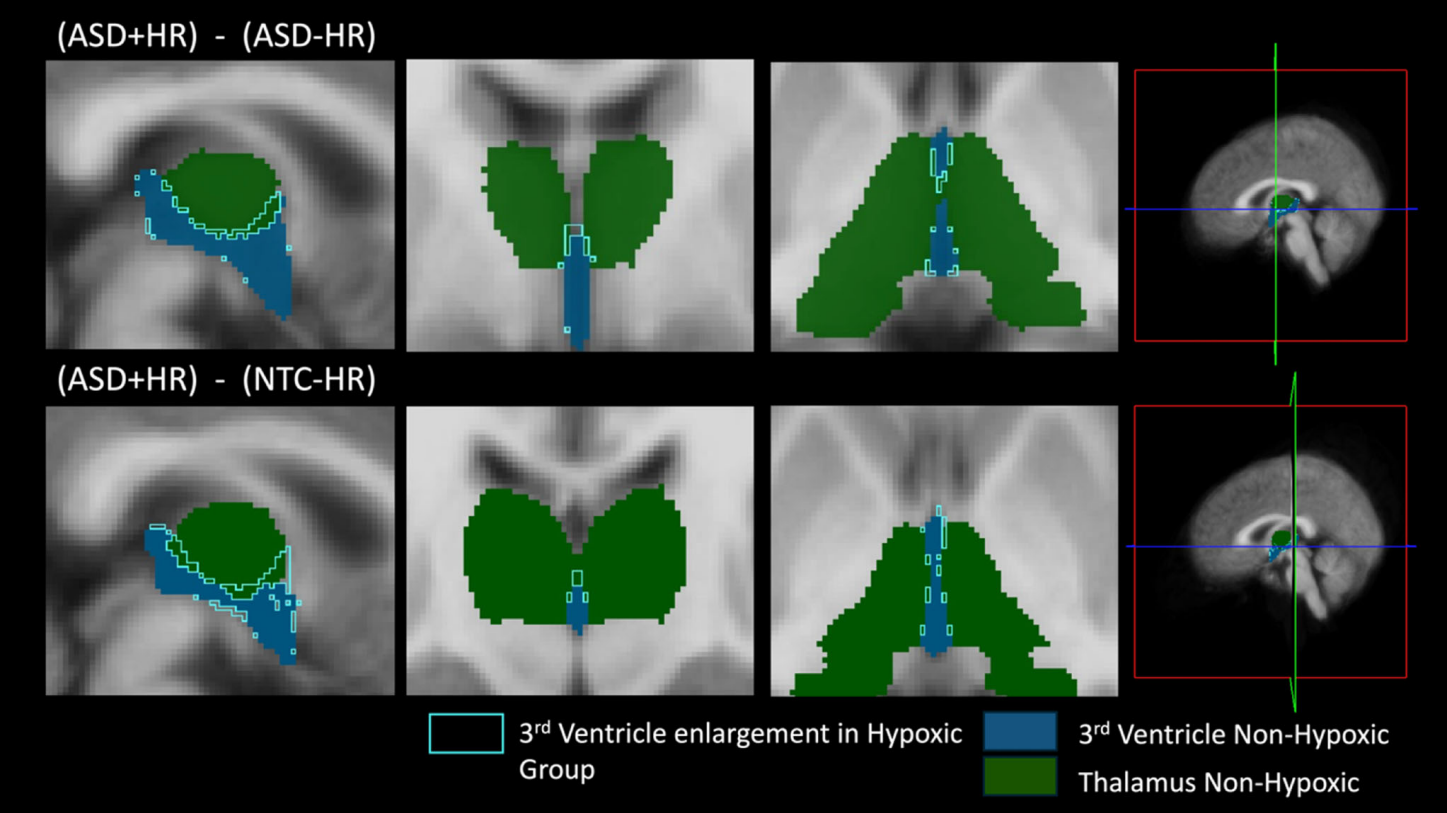
Voxel‐wise segmentation of third ventricle enlargement. Neurological view of the three planes of the voxel‐wise segmentation group difference of the third ventricle. Outlined areas in light blue were segmented by subtracting the composite image of the ASD + HR group and the nonhypoxic groups. Areas in dark blue represent the segmented nonhypoxic third ventricle and areas in dark green represent the segmented nonhypoxic thalamus obtained from the nonhypoxic composite image. ASD, autism spectrum disorder; HR, hypoxic risk; NTC, neurotypical control.

Voxel‐wise differences of ventricular volumes revealed consistent enlargement for the ASD + HR group compared with nonhypoxic groups (ASD‐HR, NTC‐HR) in the third ventricle. This enlargement of the third ventricle extended to border the ventral and anterior portions of the thalamus (see Figure 3). Notably, increased ventricular volume was positively associated with number of hypoxic risk exposures in the combined sample (*ρ* = 0.307, *p =* 0.002). We further explored whether there were group differences in participants with versus without specific hypoxic risk condition categories (i.e., preplacental, uteroplacental, and postplacental). Given that only five participants had exposure to prenatal hypoxic risk conditions in the control group, these contrasts were run within the ASD group only. Independent samples Mann–Whitney U Tests indicated that ASD participants with exposure to uteroplacental hypoxic risk conditions had significantly larger third ventricle volume (*U* = 690, *p =* 0.004). Statistically significant differences were not identified for pre and postplacental hypoxic risk exposures.

### Correlation with sleep disturbances, sensory processing, and autism symptoms

**FIGURE 4:**
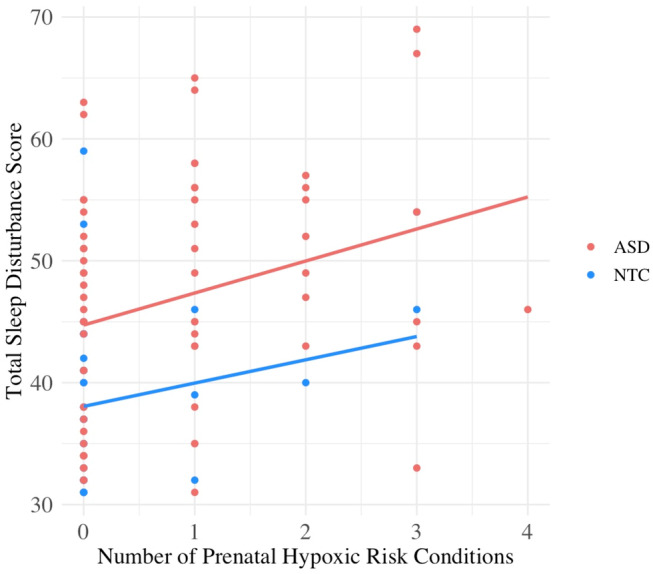
Scatter plot of total sleep disturbance and number of exposures to hypoxic risk conditions. Correlation between the number of prenatal hypoxic risk conditions and total sleep disturbance scores by group. ASD, autism spectrum disorder; NTC, neurotypical control.

Spearman's *ρ* was computed to examine the number of hypoxic risk exposures and sleep disturbance. Higher number of hypoxic risk exposures was significantly correlated with greater total sleep disturbance in the ASD group (*ρ* = 0.312, *p* = 0.010), but not in the NTC group (*ρ* = 0.215, *p* = 0.238) (Figure 4). Given the significant association in the ASD group, posthoc correlations were computed to examine associations with specific subscales of the CSHQ, including bedtime resistance, daytime sleepiness, night wakings, sleep disordered breathing, and sleep duration; however, none of these associations were statistically significant after correction for multiple comparisons.

**FIGURE 5:**
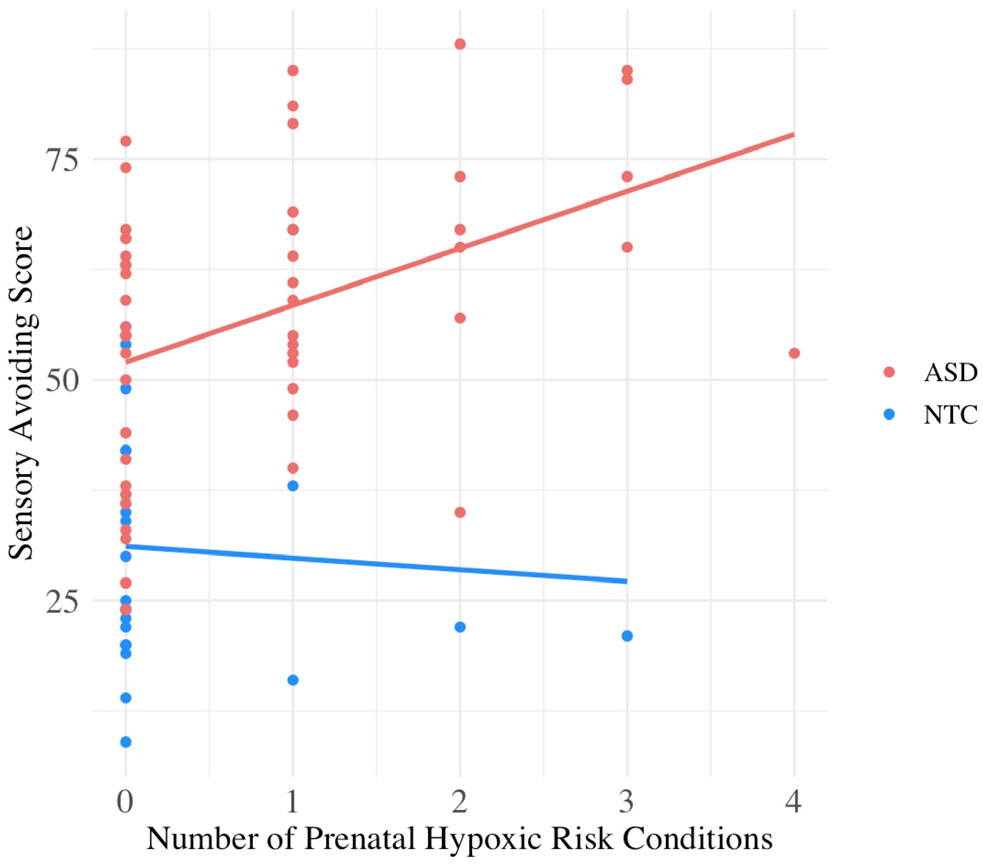
Scatter plot of total sleep disturbance and number of exposures to hypoxic risk conditions. Correlation between the number of prenatal hypoxic risk conditions and sensory avoiding scores by group. ASD, autism spectrum disorder; NTC, neurotypical control.

**FIGURE 6:**
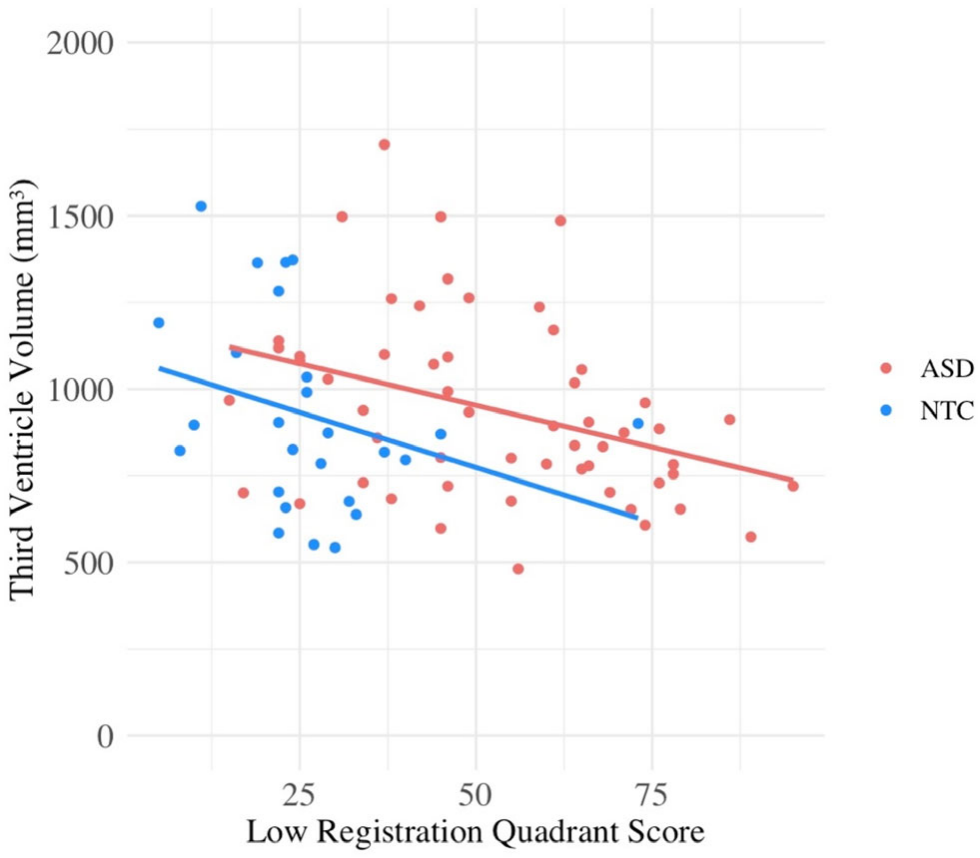
Scatter plot of third ventricle volume and low registration quadrant score. Correlation between third ventricle volume and low registration quadrant score by group. Lower score on the low registration quadrant score indicates more registration of sensory information. ASD, autism spectrum disorder; NTC, neurotypical control.

**FIGURE 7:**
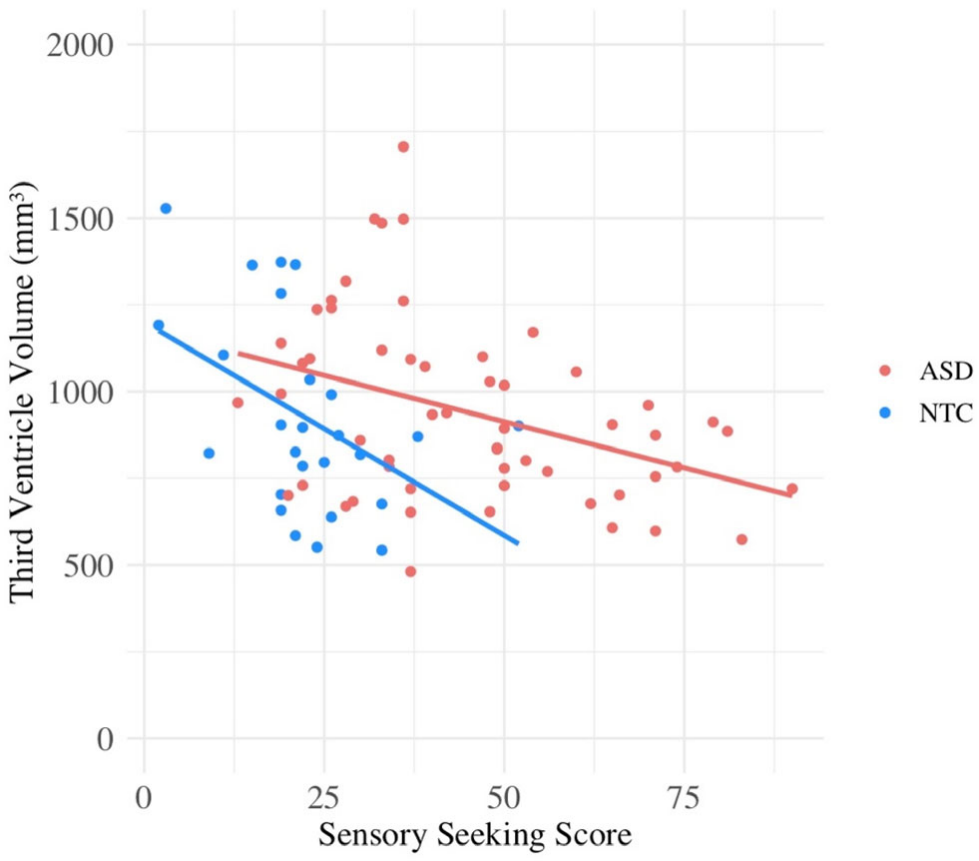
Scatter plot of third ventricle volume and sensory seeking score. Correlation between third ventricle volume and low registration quadrant score by group. ASD, autism spectrum disorder; NTC, neurotypical control.

No significant associations between sensory processing and third ventricle volume or number of hypoxic risk exposures were identified in the control group. In the ASD group however, number of hypoxic risk exposures were positively associated with sensory avoiding (*ρ* = 0.410, *p* = 0.002) (Figure 5), and greater third ventricle volume was associated with more registration of sensory information (i.e., lower score on the Low Registration quadrant), with *ρ* = −0.394, *p* = 0.003 (Figure 6), and less sensory seeking (*ρ* = −0.398, *p =* 0.003) (Figure 7). Neither group showed significant correlations between SCQ scores and number of hypoxic risk exposures or third ventricle volume.

## DISCUSSION

This study investigated autism diagnostic classification and clinical symptoms in children and adolescents who were and were not prenatally exposed to hypoxic risk conditions. The significant differences identified between the incidence of prenatal hypoxic risk conditions in ASD and NTC groups support existing literature that has established hypoxia as a risk factor for neurodevelopmental disorders (De Vries & Groenendaal, 2010; Froehlich‐Santino et al., 2014). Specifically, the heightened prevalence of hypoxic risk conditions in the ASD group corroborates earlier findings by Getahun et al. (2017), who found similar results in a large population study analyzing medical records over 18 years. The heightened prevalence puts forward a potential environmental etiology warranting further investigation.

It should be noted, however, that in these studies prenatal hypoxia is observed as an associative factor rather than a cause of ASD. While hypoxia during critical periods of gestation can be detrimental and can exacerbate neurodevelopmental outcomes, its cascade impact is nonspecific and highly variable. This distinction is supported by the multifactorial nature of ASD, where no single environmental factor has been found to conclusively cause the condition across populations. Prenatal hypoxic risk conditions, therefore, may be associated with ASD due to its impact on neural development pathways that are already vulnerable in individuals who later present with ASD. This association suggests that while hypoxia may intensify specific ASD symptoms or contribute to the variety of ASD presentations, it operates within a preexisting framework of genetic and environmental factors that collectively contribute to the development of ASD. This perspective reframes prenatal care practices with heightened vigilance in monitoring environmental risk factors among pregnant mothers, especially those at risk of ASD. By acknowledging the increased presence of hypoxic risk conditions in individuals with ASD, we underscore the need for a preventative approach that might mitigate some of the modifiable risk factors associated with ASD.

The ANCOVA results affirm the impact of prenatal hypoxic risk on brain morphology, specifically that ASD + HR youth exhibit larger third ventricle volumes. Group membership based on hypoxic risk and diagnostic status was significantly associated with differences in ventricular volume. The increase in size concurs with prior studies reporting that hypoxic damage in ventricular areas leads to increased ventricular size (Minowa et al., 2017). Our findings also highlight the enlargement of the third ventricle, consistent with previous findings of the ventricles as a region sensitive to hypoxic injury in preterm and full‐term neonates (Huang & Castillo, 2008). Given the susceptibility of the thalamus to damage as a result of prenatal hypoxic risk exposure (Okereafor et al., 2008; Pasternak et al., 1991), the selective enlargement of the third ventricle may be secondary to thalamic volume loss as an ex vacuo phenomenon. Future investigations could be conducted to determine if specific types of prenatal hypoxic risk conditions have differential effects on specific thalamic nuclei. Furthermore, since the third ventricle is encased within surrounding structures, its enlargement may cause or reflect disruption to adjacent systems. Previous research has suggested that the third ventricle's enlargement may have an impact on surrounding regions like the hypothalamus and thalamus (Wolfe et al., 2015). These regions are implicated in the regulation of sleep and wakefulness, which could potentially lead to the observed sleep disturbances (Gent, Bandarabadi, et al., 2018; Gent, Bassetti, et al., 2018; Magnin et al., 2010). Furthermore, pairwise comparisons revealed specific group differences in ventricular volume between the ASD + HR and ASD‐HR groups, as well as between the ASD + HR and NTC‐HR groups. This suggests that hypoxic risk conditions are a contributing factor to the observed third ventricle enlargement in comparison to nonhypoxic groups. Although we acknowledge the multifactorial nature of sleep disturbances, when focused on the significant enlargement of the third ventricle in the ASD + HR group, it is plausible to propose that this structural change could influence the functionality of adjacent regions and thereby impact sleep.

The significant correlations observed between the number of prenatal hypoxic risk conditions and the CSHQ total sleep disturbance scores corroborate these findings and add another layer of understanding to the effect of ventricular enlargement in thalamic regions. The positive relationship implies that increased exposure to prenatal hypoxic risk conditions may exacerbate sleep problems in this population. For example, one potential mechanism by which hypoxic risk may exacerbate sleep problems is via thalamic injury (leading to the identified ventricular enlargement) and its subsequent impact on thalamic connectivity. Specifically, the thalamus, integral to sensory processing and sleep regulation, shows reduced functional communication with the neocortex in individuals experiencing sleep disturbances (Jan et al., 2009; Picchioni et al., 2014). Click or tap here to enter text.

Furthermore, there is growing evidence of the involvement of the anterior thalamus in REM sleep, non‐REM sleep, and wakefulness (Kaufmann et al., 2006; Simor et al., 2021; Szabó et al., 2022; Zou et al., 2021). The third ventricle enlargement identified in the ASD + HR group of the current study extends to regions anterior, posterior, and ventral to the thalamus (see Figure 4). Thus, the association between sleep disturbance and number of hypoxic risk exposures in our sample may be due to structural abnormality (reduced size) of the anterior thalamus and corresponding impact on sleep–wake transitions. Moreover, hypoxic damage to thalamic structures could also have an indirect impact on sleep problems via impact on sensory processing. Indeed, we also identified significant associations between sensory dysfunction and both number of hypoxic risk exposures and third ventricle volume. Notably, the nature of sensory dysfunction identified in these associations is suggestive of a pattern of sensory overresponsivity (i.e., increased sensory avoidance, increased registration of sensory information, and reduced sensory seeking). Sensory sensitivity has been associated with sleep problems in autism (Hollway et al., 2013; Manelis‐Baram et al., 2021; Mazurek & Petroski, 2015; Reynolds et al., 2012), and growing evidence suggests that this sensory dysfunction contributes to sleep problems via differences in thalamic structure and thalamocortical connectivity. For example, sensory sensitivity and functional overconnectivity between the thalamus and auditory cortex have been linked to sleep problems in ASD (Linke et al., 2021). In this study increased BOLD signal in auditory cortex during sleep was also reported relative to a control group. The authors suggested that a failure of auditory response suppression during sleep may contribute to sleep problems for autistic individuals. This hypothesis is theoretically motivated by literature characterizing the interaction of the sleep and sensory systems (Andrillon & Kouider, 2020; Andrillon et al., 2016; Chen et al., 2016; Velluti, 1997); however, no study to date has examined differences in cortical auditory response while awake versus during sleep in autism. Taken together, this set of evidence suggests a complex interplay between prenatal environmental factors, thalamic connectivity, sensory processing, and sleep architecture that warrants further investigation via studies that directly investigate thalamic functional connectivity in individuals with ASD who have been exposed to prenatal hypoxic risk conditions. These findings present another area of clinical significance, potentially guiding targeted interventions that point to hypoxia‐related sleep disturbances.

These results should be considered in light of certain limitations. First, the study's design relies on parental reports and introduces potential recall bias. Future research may benefit from prospective longitudinal designs that can track developmental trajectories from prenatal exposures to later life outcomes using specific biomarkers. Another limitation is the small sample size in the neurotypical control group with prenatal hypoxic risk exposure (NTC + HR, *n* = 5). This limited sample size makes it difficult to draw conclusions about the differences in ventricular morphology attributable to hypoxic risk exposure more broadly, versus specific vulnerability in the ASD group. Future studies should aim to include a larger sample size for this group to determine if there is a difference in the impact of hypoxic risk exposure in ASD versus NTC groups. Further, while composite images provide valuable visual evidence of group‐level voxel wise differences, longitudinal studies will be essential to quantify changes in ventricular size over the course of development.

## CONCLUSION

In conclusion, the current study contributes to a growing body of evidence highlighting prenatal hypoxic risk conditions as significant variable risk factors in the neurodevelopmental profile of individuals with ASD. Our findings indicate a heightened incidence of prenatal hypoxic risk conditions and increased third ventricular size in individuals with ASD compared with NTC youth. Further, these findings were associated with sensory overresponsivity and increased sleep disturbances, suggesting that these prenatal environmental risk factors can have significant effects on neurodevelopment and behavioral outcomes in youth with ASD. These findings hold promise for a better understanding of the role of prenatal exposure to hypoxic risk conditions on thalamic injury, ventricular developmental, sensory dysfunction, and sleep problems. They emphasize the need for targeted strategies that anticipate and address the effects of hypoxic risk conditions, ultimately leading to improved health outcomes in affected individuals.

## FUNDING INFORMATION

This research was supported by the National Institutes of Health (grant numbers R01HD051747‐01A1, K23DC016637‐01A1, and R01DC019167‐01A1), Autism Speaks Royal Archmasons Central Auditory Processing Disorder Awards (11637), and UCSF Weill Institute for Neuroscience Weill Award for Clinical Neuroscience Research (2016038) awarded to CD.

## ETHICS STATEMENT

This study was approved by the UCSF IRB (#21‐33613: UCSF Speech, Voice, & Communication Study, and #11‐05249: Magnetic Source Imaging of Speech and Language Processing).

## Data Availability

The data that support the findings of this study are available from the corresponding author upon reasonable request.
